# Supplementary material for: Direct observation of the Dirac nodes lifting in semimetallic perovskite SrIrO3 thin films
Source: Sci Rep. 2016 Jul 26;6:30309. doi: 10.1038/srep30309 (PMC4960618; doi:10.1038/srep30309)
Supplement: Supplementary Information [file srep30309-s1.doc]

***Supplementary Information for* Direct observation of the Dirac nodes lifting in semimetallic** **perovskite SrIrO_3_** **thin films**

Z. T. Liu^1^, M. Y. Li^1^, Q. F. Li^2,3^, J. S. Liu^1^, W. Li^1^, H. F. Yang^1^, Q. Yao^1,4,5^, C. C. Fan^1^, X. G. Wan^2^, Z. Wang^1^ and D. W. Shen^1,6,*^

^1^State Key Laboratory of Functional Materials for Informatics,

Shanghai Institute of Microsystem and Information Technology (SIMIT),

Chinese Academy of Sciences, Shanghai 200050, China

^2^National Laboratory of Solid State Microstructures and Department of Physics,

National Center of Microstructures and Quantum Manipulation, Nanjing University, Nanjing 210093, China

^3^Department of Physics, Nanjing University of Information Science &Technology, Nanjing 210044, China

^4^State Key Laboratory of Surface Physics, Department of Physics, and Advanced Materials Laboratory, Fudan University, Shanghai 200433, China

^5^Collaborative Innovation Center of Advanced Microstructures, Fudan University, Shanghai 200433, China

^6^CAS-Shanghai Science Research Center, Shanghai 201203, China

^*^dwshen@mail.sim.ac.cn

1. **Thin films characterizations.**

Since IrO_2_ octahedra are rotated around the *c* axis and tilted around the [110] axis, SrIrO_3_ has an orthorhombic perovskite structure, and one orthorhombic unit cell contains four formula units with the space group of *Pbnm*^1^, as illustrated in Figure S1 (a). Figure S1 (b) shows the RHEED specular beam intensity oscillations as a function of time along the [100] azimuthal direction. Such oscillations can be observed through the whole course of the 25 unit-cell thick film growth, demonstrating the persistent layer-by-layer growth mode. Besides, since all growths were terminated after an integral number of oscillations, we expect the IrO_2_ termination for all films. The insets of Figure S1 (b) show the typical RHEED patterns of the starting SrTiO_3_ (001) substrates and SrIrO_3_ films after growth. The streak patterns demonstrate the atomic flatness for each layer during the epitaxial growth, which guarantees the quality of our photoemission spectra. The crystallographic quality of these films was then checked by the X-ray diffraction *θ-*2*θ* scan [Figure S1 (c)], in which the persistent Keissig fringes around both the (001) and (002) Bragg reflection peaks indicate the high quality of the films. Moreover, our Laue fitting of these fringes indicates a film thickness of 25±0.5 unit cells (not shown), which is in good agreement with the value by counting the RHEED oscillations. Fig. S1 (d) is a dark field high resolution TEM (HRTEM) image of orthorhombic SrIrO_3_ films, we can see that the interface is atomically sharp and SrIrO_3_ films is quite uniform which implies that our SrIrO_3_ films only have a single phase (orthorhombic phase).


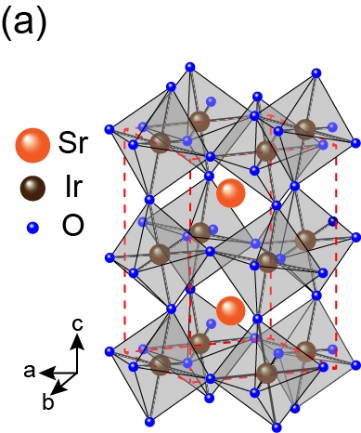

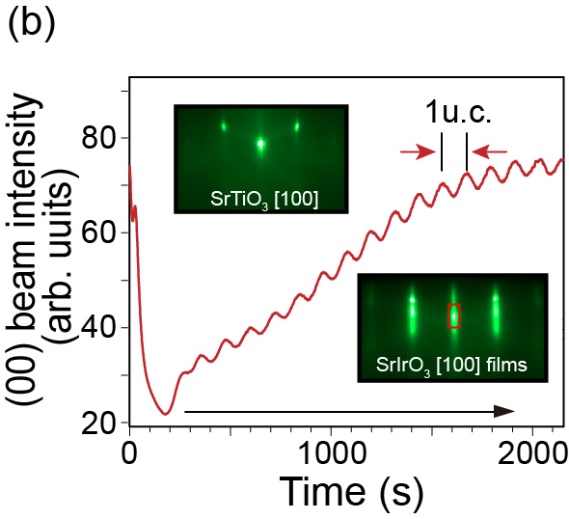


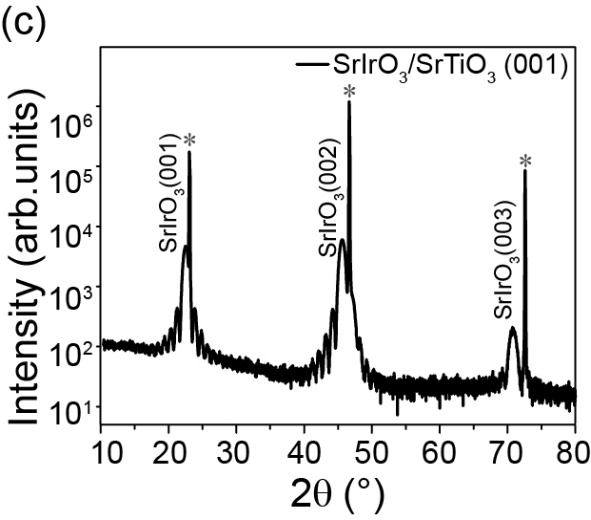

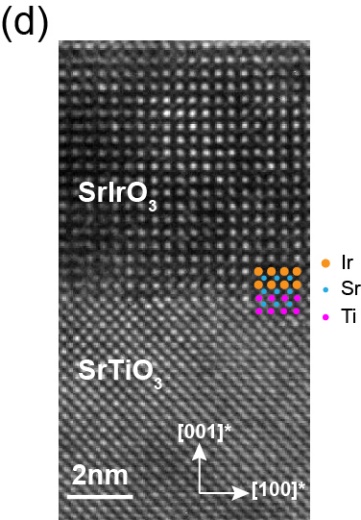


**Figure S1| Crystal structure and films characterizations.** (a) The representative unit cell of orthorhombic perovskite SrIrO_3_. Note one unit cell contains four chemical formula units. (b) RHEED intensity oscillations as a function of growth time for a typical 25 u.c. SrIrO_3_ film. Insets include typical RHEED patterns for the SrTiO_3_ substrates and the SrIrO_3_ films. (c) The XRD 2*θ* scan for the 25 u.c. SrIrO_3_ films. (d) The high resolution TEM micrographs of SrIrO_3_ films grown on SrTiO_3_ substrates.

1. **Details of density functional theory calculations.**

Density functional theory (DFT) calculations were performed using the Vienna ab-initio simulation package (VASP) code^2,3^. The valence and core interactions were described by the projected augmented wave method^4^. We also investigated the effected of strong SOI on the electronic structure. We use the experimental lattice constant, and optimize the internal atomic coordinates until the corresponding forces are less than 0.01 eV/Å.

**References**

1. Zhao, J. G. *et al*. High-pressure synthesis of orthorhombic SrIrO_3_ perovskite and its positive magnetoresistance. *J. Appl. Phys.* **103**, 103706 (2008).
2. Kresse, G. & Furthmuller, J. Efficient iterative schemes for ab initio total-energy calculations using a plane-wave basis set. *Phys. Rev. B* **54**, 11169 (1996).
3. Kresse, G. & Furthmuller, J. Efficiency of ab-initio total energy calculations for metals and semiconductors using a plane-wave basis set. Comput. Mater. Sci. **6**, 15 (1996).
4. Kresse, G. & Joubert, D. From ultrasoft pseudopotentials to the projector augmented-wave method. *Phys. Rev. B* **59**, 1758 (1999).
